# International medical students’ perspectives on factors affecting their academic success in China: a qualitative study

**DOI:** 10.1186/s12909-022-03597-z

**Published:** 2022-07-27

**Authors:** Qinxu Jiang, Hugo Horta, Mantak Yuen

**Affiliations:** 1 Xuzhou Medical University, Yunlong District, Xuzhou, China; 2 Social Contexts and Policies of Education, Faculty of Education, The University of Hong Kong, Pokfulam Road, Hong Kong SAR, China; 3 Center for Advancement in Inclusive and Special Education, Faculty of Education, The University of Hong Kong, Pokfulam Road, Hong Kong SAR, China

**Keywords:** Medical students, Medical education, International students, China, Academic success, Students’ perspectives, Qualitative study

## Abstract

**Background:**

The number of international students who choose China as their destination for quality medical education is rising, particularly those from developing countries, but little is known about their adaptation and educational experiences at Chinese universities. This study explored the factors that these students perceived to have influenced their academic success.

**Methods:**

Semi-structured interviews were conducted with international students (*N* = 40) from developing countries from September 2020 to January 2021. Participants were graduates or in their second, third, fourth, fifth, or sixth academic year in two university medical schools. Interviews were audio-recorded, transcribed, and analyzed using a thematic analysis approach.

**Results:**

The participants chose China to study medicine based on cost, teaching resources, quality of medical education, recommendation, and safety factors. They considered an increase in medical knowledge, clinical skills and communication skills as an indicator of academic success. Positive factors affecting academic success were the support system (family, friends, seniors) and campus resources (library, laboratories, extra-curricular activities, scholarship). Negative factors were (i) issues affecting learning (English language barrier), adjusting to the medical education system in China, learning difficulties, failing exams, internship difficulties, problems with online learning during the pandemic, (ii) sociocultural issues (lacking knowledge of the Chinese language, challenges in daily life, perceived discrimination, interpersonal relationships), (iii) wellbeing issues (physical and mental health issues), and (iv) other challenges (climate, food, finance, scholarship). The influence of teachers, administrators and classmates was perceived as both positive and negative.

**Conclusions:**

Factors affecting the academic success of international medical students at Chinese universities are multi-faceted. It is the collective responsibility of the host society, universities, teachers, administrators, classmates, families, and students themselves to address these factors in order to support and help students achieve academic success. Findings in our study support recommendations to improve teachers’ English language skills and pedagogy and to invest in administrators’ professional development. They also suggest that greater awareness of students’ sociocultural and mental challenges and optimizing the positive influence of classmates could strengthen student support and better address student academic difficulties. The English proficiency and prior academic performance of international students should be considered during recruitment. Given the rapid growth in international MBBS programs in China, further research on the experiences of international students in China’s medical programs is needed.

## Background

By 2019, China had become the largest receiving country for international enrollments in Asia and the third largest worldwide after the U.S. and UK [1]. China has close to 500,000 international students from 194 countries studying in 1,004 universities. Most of these students come from developing countries in Asia (60%) and Africa (17%) [2]. Since 2006, Western medicine has been the most popular subject for international undergraduates coming to Chinese universities, and by 2018, 52,946 degree-seekers out of 55,225 international undergraduates were studying Western medicine. An overwhelming proportion of these international students were studying in English-medium Bachelor of Medicine and Bachelor of Surgery (MBBS) programs. Degree-seekers who were granted Chinese government scholarships, excluding provincial government scholarships, accounted for only 8% of the total population of 55,225. This means that an estimated 9 out of 10 international MBBS students were self-fee-paying. The majority of international MBBS students came from developing countries in Asia and Africa and were self-sponsored.

Universities authorized by the Chinese Ministry of Education (MOE) to recruit international MBBS students are recognized by the Medical Council of China and the World Health Organization (WHO) in the directory of World Medical Schools. According to the MOE, the MBBS curriculum in universities and colleges is mostly similar. The international MBBS program in China is a six-year program taught in English, and its curriculum consists of five years’ pre-clinical (Chinese language, liberal arts, natural science and basic medical science subjects) and clinical subjects and a one-year internship.^1^ The training mode and curriculum provided by the international MBBS program in China are similar to those provided to Chinese medical students [3]. However, medical universities have the autonomy to design their own courses, which may lead to differences in terms of curricular and learning demands, which may not meet international standards [4]. Rotational internship can be conducted in either designated hospitals in China or the students’ home country depending on students’ own wishes. Typically, there is an academic responsible for MBBS student affairs and teaching, and international students and their Chinese counterparts have separate classes.

For international students, particularly those from developing countries, studying medicine in China is expected to brings benefits both for themselves and for their countries of origin [5]. Students have the opportunity to obtain a medical degree, an opportunity they may be denied in their home country because of academic competition, the limited number of places, financial constraints, corruption, or other reasons [5]. These students, upon graduation, are likely to become an important addition to the healthcare workforce in their home country, where human resources for healthcare may be in strong demand [6, 7], or they may practice medicine in other countries [8, 9]. Chinese universities also benefit, reaping reputational and financial gains by hosting international students [5, 6]. In hosting international students, Chinese medical universities contribute to national policy objectives, such as the *One Belt One Road Initiative,* by advancing the internationalization of Chinese higher education, building world-class universities, promoting cross-cultural transfer of knowledge and mutual learning, cultivating national soft power, and expanding trade in educational services [6, 10].

Generally, international students are recruited based on high school transcripts as proof of academic ability, without entrance exam or interviews, while proof of English language ability, such as IELTS or TOEFL is not a mandatory requirement [11–13]. Currently, enrollment channels are relatively simple, mainly relying on introductions by intermediaries, such as educational agents [3, 11, 12]. In contrast to the enrollment procedures of receiving developed countries, China’s admission system for accepting international students lacks a unified comprehensive evaluation mechanism for student enrollment, resulting in loose admission thresholds for international students and an uneven quality of international student enrollment [4, 12].

To enure that MBBS programs meet international standards, the MOE has introduced rules and regulations to this effect. One of these regulations is that the MOE decides to assign to universities fewer and a more dynamic quota of places based an annual evaluation of the quality of each institution’s MBBS program, with the highest number of places up to 100, followed by 60, 50, 40, or 20. However, despite these efforts to ensure the quality of MBBS programs, the academic outcome appears to be discouraging. For example, Indian medical graduates who have obtained an MBBS in China need to pass the Foreign Medical Graduates Exam (FMGE) in order to obtain a license to practice in India^2^ [14]. According to the latest report from the India National Board of Education, from 2015–2018, the FMGE pass rate of Indian medical graduates with an MBBS from China was only 11.9%; in 2019, it was 27.14%; and in 2020, it was 12.92% [15]. In 2021, the passing rate of Indian graduates from China was only 13.28%, lower than that of Indian graduates studying MBBS programs in other countries: Bangladesh (38.68%), Ethiopia (35.71%), Belize (32%), Cuba (23.07%), Belarus (19.13%), Georgia (18.79%), Ukraine (17.09%), and Armenia (14.63%) [16]. Similarly, from 2016 to 2018, the average Nepal Medical Council licensing exam pass rate of Nepalese medical graduates with an MBBS from China was also very low, as only one third of the graduates passed it (34.33% compared with higher graduation rates of national graduates; [17]). Considering that China has the largest number of Indian students compared with other countries, the low FMGE pass rates raise doubts about the quality of China-educated candidates, and China’s MBBS programs [18].

Academic success is the top priority of international students studying in China, especially for degree seekers [19, 20]. This is because academic grades and a degree matter significantly in students’ prospective postgraduate education or career development and are known predictors of prospective socioeconomic success [21]. Academic performance in medical school is important for success in licensing exams and can predict workplace performance [22]. Fee-paying international students weigh up the value of their payment to study in China. Their learning experience is ultimately likely to influence their recommendation of a particular Chinese institution to their parents, relatives, friends or peers [23, 24], which may affect China’s long-term international education development. Therefore, facilitating the academic success of international students should be a vital task for Chinese higher education.

Existing research conducted in contexts outside China stresses specific factors determining the academic success of international students. English language proficiency is widely believed to be highly correlated with academic success although the results of studies concerning the correlations between TOEFL, IELTS, GMAT tests scores, self-rated English language ability and GPA have actually tended to yield inconsistent findings [25–27]. Another factor affecting academic success and completion of studies is a student’s financial resources [28, 29]. When international students fall short financially, it can be a source of stress [30, 31]. Interrelated factors also affecting students’ mental and emotional well-being include adjustment to sociocultural differences, academic demands and psychological adjustment [32]. Students need to develop coping strategies to address these issues [33] and need to draw on campus resources such as the library, academic advice, counseling, the writing center and financial aid [30, 34, 35]. Problems are fewer in institutions with qualified and culturally-sensitive teachers [20, 36]. In terms of students’ own attributes, the rate of success is higher when students have academic self-efficacy and motivation [37, 38], good prior academic performance [39], positive attitudes towards learning [34] and effective study habits [40], and are able to learn independently.

The above findings concerning academic success are mainy based on data from Western settings. At the present time, little is known about the academic success of international medical students studying in a China setting, and findings from the West may not be generalizable to China [41]. Moreover, a better understanding of why international students choose Chinese medical universities to study, and of students’ perceptions of academic success and the factors influencing their academic success or failure is important not only for the students themselves but also for Chinese universities in order to provide a better service for these students, improve the teaching, and contribute to the global development of the medical profession. It is therefore timely to investigate the factors that international medical students perceive to be relevant to their academic success in a Chinese university setting. The aim of this study was thus to explore these factors with a sample of successful and unsuccessful international medical students from two medical faculties in China by means of individual interviews. The findings should enable such faculties to develop better policies and practices in order to enhance the academic success of this group of international students. The study had three overarching research questions:

- RQ1. What are participants’ reasons for choosing China as a country in which to study medicine?
- RQ2. What are participants’ definitions of academic success as a medical student?
- RQ3. What factors do participants perceive as influential in their academic success?

## Method

### Participants

Participants were selected from two universities offering medical studies in Jiangsu Province in east China. The two institutions followed the same MBBS curriculum, and their students had similar characteristics in terms of country of origin and age. As the purpose of the study was to explore factors that may influence international MBBS students’ academic success, it was deemed appropriate to involve participants with extremely high and low grades in order to learn as much as possible from the cases [42]. To this end, extreme case sampling, which is used to select cases that are “unusual or special in some way, such as outstanding successes or notable failures” (42, pp. 230–231), was used for participant recruitment. Two groups of students, one containing ‘outstanding’ students and the other ‘poor achievement’ students (based on their academic grades), were recruited with the help of staff at the two universities. Second and sixth year students as well as graduates were targeted as, unlike first year students, they had received academic grades. Ultimately, a total of 40 students were interviewed: 18 high-achievers (H) and 22 low-achievers (L). This sample was deemed adequate because saturation of data was achieved [43]. Most of the participants were from India (*n* = 16), Bangladesh (*n* = 7), Nigeria (*n* = 4), and Sri Lanka (*n* = 3). Eighteen participants were male and twenty-two were female (Table 1).

**Table 1:** Participant profile

	**University A**	**University B**	**Total**
**Participants **	27	13	40
**Male**	11	7	18
**Female**	16	6	22
**High achievers **	12	6	18
**Low Achievers **	15	7	22
**Country of origin **			
India	10	6	16
Bangladesh	7	0	7
Nigeria	4	0	4
Sri Lanka	0	3	3
Australia	0	1	1
Thailand	0	1	1
Egypt	1	0	1
Comoros	1	0	1
Tanzania	1	0	1
Botswana	1	0	1
Ghana	0	1	1
Zanzibar	0	1	1
Zambia	1	0	1
Zimbabwe	1	0	1
**Academic year**			
2^nd^ year	2	2	4
3^rd^ year	2	2	4
4^th^ year	2	2	4
5^th^ year	5	3	8
6^th^ year	6	2	8
Graduates	10	2	12

### Data collection

Semi-structured interviews were used to collect data. However, due to the COVID-19 outbreak in January 2020, some participants were still outside China and unable to return as a result of border restrictions. For these students, individual virtual interviews were scheduled. For face-to-face interviews with the other students, a comfortable and quiet spot on the university campus was arranged to make the interviewees feel at ease to talk. All interviews were conducted in English and audiotaped by the first author. Each interview lasted between 40 and 80 min.

### Data analysis

All audio recordings were transcribed verbatim into text, and the material then subjected to thematic analysis [44]. The researchers first familiarized themselves with the material, searched for themes, and generated initial codes, and then, as the analysis progressed, refined and named the themes. Several approaches were used to address the trustworthiness and validity of the analysis procedures: member checking, peer debriefing, expert reviewers, and reflective journaling.

### Ethical approval

Ethical approval was obtained from the Ethics Committee of University A prior to the commencement of the study. The purpose, procedures, and time implications of the study were explained to the participants before the interviews started, and the interviewer answered participants’ questions or concerns. The participants were guaranteed anonymity and confidentiality. Their participation was voluntary, and a written consent was collected from all students before the interviews started.

## Results

### Main themes resulting from the data analysis

Data from the interviews were organized into four main themes: (1) motivation for choosing China to study medicine, (2) definitions of academic success, (3) factors positively affecting academic success, and (4) factors negatively affecting academic success. In each theme, specific sub-themes were derived from the analysis of participants’ responses.

### Theme 1: Motivation for choosing China to study medicine

#### Theme 1.1: Push factors

The majority (52.5%) of participants mentioned that medical education was more expensive in their own countries than in China. They also said that gaining admission to medical school in their home country was a fiercely competitive process. A few (10%) even suggested that, in their country, corruption seemed to be involved in obtaining a place at medical schools. High costs coupled with a perception that admission was often not based on merit had pushed them to seek alternatives elsewhere. The low cost and the availability of internationally recognized MBBS programs in Chinese universities made these courses attractive to them:

I was trying to do an MBBS in my country, but due to a lack of places and the cost in India, it is very expensive. (M, H, 4^th^ year, India).

Other push factors in participants’ choice of China as a country in which to study medicine included a shortage of laboratory and teaching resources and the long duration of MBBS programs in their country of origin. As one participant commented,

Back home we don’t have enough teachers. Imagine that if I get placed in a class of 200 or 500 and you don’t even get to learn properly because the teacher is managing to try to teach the whole class. (F, L, 4^th^ year, Zambia)

### Theme 1.2: Pull factors

Participants indicated that there were many advantages of studying medicine in China. The majority (55%) of participants said that they had chosen China because of strong recommendations from their family members, relatives, friends, and seniors. Their choice of China was often attributable to perceptions of the country’s high level of health care, use of technology in treating patients, and the high quality of medical education in China:

I thought about selecting an Asian country. My dad travelled to China before and liked the hospitality there. So, with this recommendation, with some of teachers’ recommendations, I selected China because of the friendly environment and the quality of the education in the country. (F, H, 3.^rd^ year, Sri Lanka)

Chinese culture and language seemed appealing factors to those who were interested in gaining such experiences. These were perceived as an added value by some (37.5%) of the participants. Participants were also attracted to China by the fact that it was a friendly and safe society, especially for girls. The two quotes below exemplify both sentiments:

From my childhood, I have heard about China being a really beautiful country. And it also has a different culture especially the lifestyle. I was interested in Chinese, Chinese culture, history, and I used to read stories related to China and also watching Chinese movies…I wanted to experience how it would be in China. (M, L, 5^th^ year, India)

One of my cousins who was studying in Nanjing in China recommended that I come here (China)... It’s also a safe country. (F, H, 3^rd^ year, Zimbabwe)

### Theme 2: Definitions of academic success

The overwhelming majority (92.5%) of participants identified medical knowledge, clinical skills and communication skills as the most important elements of learning that must come from the MBBS. This was a prevalent sentiment among the participants:

Initially, when I was doing the MBBS, I felt grades and doing academically well was important, but as we graduated, we felt that getting medical knowledge was more important and treating patients was more important than our grades. What we learned through those five years will guide us for the rest of our lives other than grades and all. (F, H, graduate, India)

In hospital, patients won’t come and check your degree and your marks: ‘show me your marks’. They will ask you for the treatment…maybe a patient is uneducated, so communication with the local patients in the local language or local signals matters most. (M, L, graduate, India)

However, although the practical knowledge acquired and the attitudes developed were relevant, other participants acknowledged the importance of academic grades as a reflection of how well one had learned and a determinant of MBBS graduation:

Good marks are important because they show how much we understand the subjects. Fewer marks mean we have doubts on that subject. If we have good marks, it means we have good knowledge in theoretical manner on that subject. (F, H, 5.^th^ year, Sri Lanka)

### Theme 3: Positive factors

#### Theme 3.1: Support system

All the participants agreed that social support from family, friends, peers, seniors, and even others with the same nationality played a significant role in helping them overcome difficult times regarding academic, sociocultural, emotional wellbeing, and other challenges in China. Social support was a critical and useful coping strategy utilized among students. Emotional support from family, and financial support to purchase learning materials, friends’ support, and peers’ and seniors’ support for daily life events such as cooking, shopping, illness, pandemic anxiety, loneliness, learning, and preparing for exams contributed positively to student’ academic studies, social life, daily life and general wellbeing. It is important to note that this support came not only from friends and relatives in China but also from those in the students’ country of origin. A case of social support in China is demonstrated by the quote below:

The sudden death of my father led me into depression. My friends (at University A) all supported me in getting out of it and forgetting it. They said, ‘we are here for you. You can ask for help at any time.’ They used to come to my dorm to talk and take me out. With their help, I slowly came out of depression. (F, L, 5^th^ year, Bangladesh)

### Theme 3.2: Campus resources

Campus resources were important supplementary support for students’ lives and learning. The majority (72.5%) of the participants pointed out that the university library, with English books, other facilities, and a conducive learning environment, was one of their favorite places to go. Participants expressed their opinion that hands-on laboratory classes helped them to understand concepts better. They added that being allowed to use laboratory equipment after class was tremendously beneficial. The same type of benefit was classrooms that stayed open to students when they wanted to study. For example, one student commented:

I think the most important resource provided by the university is definitely the library. It gives me the ambiance. It is very quiet and spacious. I borrowed many medical books to read...It saved me money from purchasing hardcopy textbooks. (F, H, 6^th^ year, Nigeria)

Extracurricular activities, both academic (e.g., International Physiology quizzes, Chinese Language contests) and non-academic (e.g., sports meetings, cooking, drama, singing or dancing competitions), were very popular among participants (42.5%) and perceived to be positive for their mental health. These activities helped students relax from their studies and maintain a balance between study and entertainment. Participating in activities helped them gain self-confidence and develop socially:

The drama contest helped me a lot because it brought people together. You had to talk to someone you had never talked to before. You had to speak in public and perform. That helped with the whole courage. It is very important for mental health because you had fun and you felt refreshed. I made a few friends during the drama activities. They helped me to prepare for exams.” (M, L, graduate, Nigeria)

The availability of a university scholarship not only was a motivating factor for participants but also helped those who experienced financial difficulties (from the beginning of their studies or during their studies). Good university physical infrastructure such as lakes, gardens, and outdoor seating created a peaceful environment for students in which they could relax and socialize. They perceived this to be good for their physical and mental health. In addition, the campus playground, cricket ground, football field, basketball court, and gyms offered convenient spots for students to do sports and relax. Good hostel arrangements, with access to the internet, showers, air-conditioning, heaters, electricity, and campus canteens, the availability of Halal canteens, kitchens in dorm buildings, availability of supermarkets, bank ATMs, printing services, and coffee shops enabled students to do their work on campus and save time, especially during exam time. One participant provided a more comprehensive comment on campus resources.

At each and every corner of college is a very useful...canteen…supermarkets… shops…everything is available, and we need not go out…behind the anatomy building, there is a small lake… there are some chairs over there… it is a popular place for many people when you are not feeling mentally well, or missing parents, or having other mixed emotions. I really love to go there and sit for a few minutes, just look at the nature and come back to the dorm.” (F, L, graduate, Bangladesh)

### Theme 3.3: The positive influences of teachers, administrators and classmates

Participants indicated that their favorite teachers were those with supportive characteristics such as being friendly, approachable, engaging, passionate about teaching, prepared for class, funny, interactive, inspiring, speaking good English, able to explain complex concepts, and trying their best to communicate with students. The latter attribute was evident, for example, in their clear explanations of OHP slides and use of clinical cases to teach. The good communicators had a deep knowledge of the subject matter, were able to answer students’ questions, took into account different students’ levels of ability and assigned appropriate homework. Unfortunately, effective teachers, according to participants, were in the minority. The most effective teachers also helped students prepare for exams, used the blackboard for teaching demonstration, showed visuals, and used frequent quick quizzes. They also took the trouble to learn about their students’ cultures and remember students’ names, and were able to make students feel comfortable and interested in class. In terms of discipline, effective teachers were always strict with class attendance. Some established good contact with their students by sharing personal life stories and overseas background experience. Most female teachers were perceived to be good and easy to communicate with. The participants perceived the learning materials provided by teachers to be very helpful to their studies. One participant exemplified these teacher attributes:

The anatomy teachers, every two weeks, we have a test, we have to go prepare and come back, so you have to be ready… The way the teacher interacts with us is good. He makes small jokes and you laugh together. When you laugh with someone, it means you actually get a little bit close to that person…because of that, I like to study anatomy. (M, L, 5^th^ year, Ghana)

A small number of low-achieving participants (18%) indicated that they were able to pass certain exams because they remembered the teachers’ words in class or received teachers’ individual help with their learning difficulties. Although it applied only to a minority of low-achieving participants, this positive factor allowed these students to perform better than they would have even if they had continued to be part of the low achiever student group. One low-achiever recalled:

I think for our parasitology teacher, Mrs. Chen, she was the best. I didn’t even have to read the textbook or anything because I was always engaged, I always wrote down notes. I scored 96. It was entirely because of the teacher. (F, L, 4^th^ year, India)

Participants perceived university personnel responsible for general international student affairs (including daily life management, visa arrangements, extra-curricular activities, study, scholarship, dorms, illness and so forth) to have a positive impact on their lives, learning, and wellbeing. These personnel played various roles in providing parental support, emotional care, problem-solving, pastoral care, and discipline management. They were supportive of students’ learning, cared about their academic performance, motivated students to study hard, pushed them to attend class, checked exam halls to prevent exam misbehaviors, and supervised students’ daily behaviors. Whenever students encountered life problems, international student affairs personnel would usually be the first people they would turn to for help. They were active in discovering students’ talent and provided them with activity opportunities for relaxation and all-round development. In students’ eyes, these people helped them to take their mind off problems and concentrate on studying as well as balancing study with other things:

I am very satisfied with student affairs and teaching affairs staff. They care about us, and whatever the problem we come across, we just talk to them, and they always resolve it efficiently. So, we don’t need to worry about other stuff. You just need to concentrate more on studies. (M, H, graduate, India)

Seventy-eight percent of the participants considered that having classmates around created both a competitive and a collaborative learning environment in which they were motivated to study. They were influenced by observing others studying. In addition, classmates were a important form of support by helping each other with learning, creating group studying and preparing for exams. Classmates were considered to be important sources of information about exams, learning materials and other things. For these participants, classmates were their ‘family’, friends, helpers, study partners, and information sources:

A social life is a core foundation of everything. The students I live with have played a major role for me to do well in academic study. They introduce me to different books, or good ways of learning, motivating me to study. I think they play a major role in consoling me when I am in bad times because we all have bad times…. Because we come from different places, I get an opportunity to improve my reasoning and thinking because different places means different reasoning and different thinking. (M, H, 2^nd^ year, Tanzania)

### Theme 4: Negative factors

#### Theme 4.1: Academic challenges

##### Teachers’ English language barrier

Half of the participants found it difficult to understand teachers’ accents or poor English, and they hinted that teachers might also have trouble understanding international students’ English. Participants explained that teachers’ difficulties in understading students may have been related to the different accents of students when speaking English, as well as their different manners of expression – including wording—which differed from country to country. This communication barrier caused by the English language barrier made it difficult for students to benefit fully from lecturers. Consequently, some students became confused and frustrated in class, and even when they asked for help, they had difficulties understanding new explanations from the teachers. The teachers seemed to resort to using their native language in an attempt to explain:

Sometimes I wanted to understand something better, I would approach teachers and asked questions. But I never really got complete answers. I will say seven out of ten times, we are not really satisfied with the answers, and we really have to challenge those answers because the way teachers understood the question might not be the same way we understood the question. (F, H, 6^th^ year, Nigeria)

I remember this teacher half way during teaching, he switched to Chinese. We were shocked. (F, L, 6^th^ year, Nigeria)

Participants felt that, possibly as a result of the language barrier, some Chinese teachers lacked confidence in their teaching and became ineffective. For example, they tended to read everything on the slides without explaining, and avoided interacting with students. These behaviors further exacerbated learning difficulties that the students might be experiencing, as the following quote demonstrates:

Some teachers possess subject knowledge, but they can’t teach it because of their lack of English. We can’t learn it properly, so it’s very hard to understand complex concepts. Some teachers are a bit scared to speak in English, or they may be scared they will make a mistake, so they read the slides. It’s kind of boring to us. Reading the slides and teaching the slides is different. (M, H, 6^th^ year, Australia)

##### Students’ English language barrier

Ability in English is a key element in academic success in universities. An English language barrier was evident among both high-achievers and low-achievers. For 28% of the participants, English was a foreign or second or third language in their home country, and their command of English was weak. This issue caused learning and communication struggles. Interestingly, comments from high achievers seemed to indicate that they managed to improve their standard of English quickly, while low achievers still struggled with English, as the quote below demonstrates:

I studied in a local language in India, so when I came to China, English was a totally new language to me... It was very difficult for me. I couldn’t even read a paragraph or not even a simple English sentence. Whenever I had an exam, I was very anxious because I had to cram everything. (M, H, 5^th^ year, India)

##### Adjusting to the MBBS education system

Participants mentioned differences between the MBBS curriculum and exams in China and those in their home countries. According to them, in their home country, medical subjects were arranged in the first year of an MBBS program, whereas in China, liberal art subjects and natural science subjects (mathematics, chemistry, and physics) were the primary subjects in the first year of an MBBS program. This curriculum difference disappointed some students, who perceived that it was unnecessary to repeat what they had learned in high school. Moreover, some students were weak in mathematics, chemistry and physics, and learning them became a huge challenge. They argued that this was one of the reasons why they failed, even several times, in these exams.

I failed in math. I was thinking I was running away from mathematics by choosing medicine, because I never liked math. I thought in medicine, you did not need mathematics. But when I get here, they give me math. (M, L, 2^nd^ year, Botswana)

Participants perceived late exposure to medical clerkship and limited clinical learning as other challenges in adjusting to China’s MBBS curriculum. Due to language barriers between students and teachers, and between students and patients, hands-on patient contact and clinical experience were reduced, compared to Chinese students. The participants felt that they needed more exposure to clinical environments, where they could observe and learn from practice. They needed to apply for training and learning in a hospital environment, something they found difficult, and many felt completely unprepared when they went to the internship, which minimized their ability to learn more:

We had minimum hospital hours. It is a bit difficult to me. If we had a long duration in hospital, it will be good to apply our knowledge (F, H, 5^th^ year, Sri Lanka)

After I finished my fifth year in China, I came back to Zanzibar for internship. The minute I started my internship, I realized I did not have enough clinical knowledge. Of course, I learned those things in the class but in the practical side, it was not enough. (M, L, graduate, Zanzibar)

Adjusting to exams was considered a major challenge. A substantial number of participants were not satisfied with the way exams were conducted. One source of dissatisfaction concerned open-book exams, which were not aligned with the need-to-know things, but rather with how to find things that were already available in their handouts. It was also not the kind of exam students were used to or preferred. Another source of dissatisfaction was related to the dumbing down of their knowledge. One participant was not happy with the attitude of some teachers who would make the exams extremely easy so that they could pass. This would mitigate any issue that the teachers may have with students failing their classes but was deemed irresponsible and not what the students wanted out of their learning experience:

I think open book exams for clinical subjects might not be a good idea. I realized later. Because they just gave us one reading and we gave that exam, I think we need to learn more concepts regarding those subjects and then give a closed book exam. (F, H, graduate, India)

…the teachers just gave us the exam paper. Everyone was studying that particular part of questions and gave answers, and there was no knowledge in it. It was just a cramming. I feel very betrayed because we studied day and night, but some other students just studied those ten questions and got a 100 mark in the exam. We think it is just a waste of time... (M, H, 5^th^ year, India)

Tight exam schedules, a lack of feedback after exams, writing verbatim from textbooks or notes in exams to get a good score rather than expressing one’s own understanding, were other disappointing aspects for some participants, as the quote below shows:

In my country, in high school, you were required to answer your exam questions in your own understanding, in your own words… because teachers could really understand your way of thinking. Writing in verbatim was not allowed… Coming to China, I realize that you can’t really do that. I can understand why, mainly because of the language barrier, because it would very hard to write in your own understanding using words that someone else does not understand…I realized that if I want to write my answers in the exams in my own understanding, not using what the teachers read in the slides, or what is in the textbook exactly, I am likely to get a lower score. (F, H, 6^th^ year, Nigeria)

##### Learning difficulties and failing exams

Participants (37.5%) talked about the difficulties in learning some subjects, including natural science subjects and medical science subjects. They also faced the challenges of having to study too many subjects in a semester, something that they considered overwhelming. Equally, Chinese language classes posed a big obstacle for some participants. Learning Chinese helped them gain knowledge about China and the Chinese language but also took up so much time that they thought could have been given to medical subjects. Failing exams, repeating exams, and mastering medical terminologies seemed challenging to some low-achievers. One of the participants stated this clearly:

Some of the subjects are really hard. I don’t think they can be learned in one semester, like anatomy. We did anatomy in second year first semester. The first one was overwhelming...My head is about to explode. In the head, physiology and histology, I read about that time, everything just comes in, becomes bigger because that’s when we had the cardiovascular system, physiology and we learned it for a whole five weeks. I can tell you in those five weeks I got nothing. I couldn’t understand. (F, H, 3^rd^ year, Zimbabwe)

##### Internship difficulties

Six out of twenty participants with internship experience indicated several barriers that made learning during the internship period difficult for them. The first was the language barrier. Some of the participants were unable to communicate with Chinese patients because of their limited Chinese. Some of their Chinese teachers tried to translate for them, but they were unable to convey all the information. Most doctors in the hospitals did not have time to do so. Some participants indicated they could understand everyday Chinese language but not Chinese medical terms, making communication with the patients almost impossible:

When we follow the teachers to check wards, they use medical vocabulary in Chinese to talk to patients. This is difficult for us because we can communicate in daily Chinese words, but we don’t understand medical words in Chinese. If they have time, they will explain how to use them in English; sometimes they don’t because they are busy taking care of patients. (F, L, 6^th^ year, Thailand)

It also seems that some teachers were so busy with their own work that they sometimes paid little attention to their international interns. In some departments, Chinese female patients refused to allow male foreign interns to watch or perform checkups on them. Even diseases that are prevalent in China can be different from those in participants’ home country, but no explanation or alignment seemed to be attempted so that knowledge learned in the internship could be of practical use. All these barriers were counterproductive to students’ development of key clinical skills and communication skills. One participant commented that she intended to go back to her country to redo her internship there:

For the internship now, it’s bad because of the language. You know not all the doctors really speak English and the patients don’t want you to touch them because you don’t know anything…. That’s why it’s really not advisable to do your internship in China. You don’t really get to the practical part of it. I was planning if I graduate and will go back home to do my internship in my country because the diseases also, again, are not really the same. You also go back to your country to practically redo it if you really want to know what you’re studying. (F, L, 6^th^ year, Nigeria)

##### Online learning during the pandemic

The global COVID-19 pandemic had worsened the learning experience of the participants. Since 2020, China’s zero-tolerance for infection policy had prevented many of them from returning to school to receive face-to-face classes. As an alternative, students had to continue with online classes in the form of recorded lectures rather other livestream lecturing. Students who stayed in China also received online classes but did have the benefit of meeting teachers face to face for Q and A sessions. Despite the advantages of flexible learning time and access to videos, most participants expressed emotional fatigue, boredom, demotivation, and laziness when faced with online learning. Internet disconnection or slow speed and technical problems hindered online learning and taking online exams. Students felt that the quality of online teaching and learning was compromised:

Some of the teachers aren’t too serious about their teaching. They don’t care about the teaching quality, just for teaching and passing the time. Students are not much serious about recorded classes. (M, L, 5th year, Egypt)

Learning had become more difficult due to the lack of teacher-student interaction, late replies from teachers, unavailability of practical classes, increased workload, difficulties in understanding online lectures, and so forth. Some participants had concentration issues because of family responsibilities or other distractions. All the participants who took online classes indicated that they preferred traditional face-to-face classes, and that they would have performed better if they had been taught in a traditional classroom:

…doing the MBBS online really doesn’t make any sense. It’s just like I am watching YouTube. We need practicals, we need more interactions, and we definitely need to see what is happening. That is a huge thing. (F, L, 4^th^ year, India)

### Theme 4.2: Sociocultural challenges

Participants indicated that typical challenges facing them involved the Chinese language barrier in daily life activities such as shopping, buying tickets, going to a bank, or seeing a doctor. There was also culture shock in terms of traditions, customs, religion, food, behaviors, and time. Some participants mentioned racial discrimination due to color (e.g. cursing, taking photos or videos of the students without permission, verbal harassment) from local people. The pandemic had magnified discrimination against foreigners in China, as the following statement of a participant exemplifies:

Some old women especially, want to see why your skin is black. They feel maybe you are not taking your baths or something, so they tried to scrub your skin and say like ‘emm, why is it this dark?’ Especially during the pandemic, maybe you are trying to walk into a place and you see people covering their nose. (F, L, 6^th^ year, Nigeria)

Participants also reported socialization difficulties in terms of making friends with Chinese students. These were mostly due to the language barrier and cultural differences. However, there was also perceived social segregation among international students due to language differences and grouping by nationality, which participants referred to as “politics”. Some participants also mentioned interpersonal relationship issues with roommates, friends, and romantic partners. These sociocultural challenges could cause frustration, anxiety, loneliness, irritation, even fear among students, and negatively affected their academic performance:

I am dealing with different people, different cultures, different things. Sometimes there are things I didn’t expect to do. It comes as something new. It is not everyone who is able to accommodate. If I look at my academic transcript, the academic year when I had lowest marks, it is mostly because I didn’t have a good relationship with some friends. (F, H, graduate, Comoros)

### Theme 4.3: Wellbeing challenges

Sickness and mental wellbeing matters posed large challenges to participants’ health and study, mental health in particular. Most physical illnesses, according to participants, were not that serious and could be treated with medicine. However, falling sick during exams became tough for them, even contributing to failure in exams. Homesickness, loneliness and pandemic fear and anxiety were common and challenging for most of the participants:

During the pandemic, I am the only Indian student staying over here. It’s only me and another two juniors. There are no Indians left. I have had to stay alone from January (2020 when everyone left for home till now (September). I feel lonely and homesick. Once a year I go back, but this year due to this pandemic, I couldn’t go. I miss my parents a lot. (M, H, 4^th^ year, India)

Moreover, depression caused by unfortunate events such as losing one’s family members, roommate issues, exam phobia, academic burnout and stress, and financial stress severely affected students’ health and normal study. However, there were no professional psychological counseling services aimed at international students on campus that could have helped them to get over these issues, as recognized by most students:

Towards my 4^th^ year, I was experiencing a lot of burnout. I also felt sick because I was experiencing a lot of stress at the same time. In terms of emotional wellbeing, I wanted to talk to someone and a professional, not a friend or something. I wish there was an actual counselor or like a psychologist, on the ground that at least we got to see from time to time and get some help, but there wasn’t really any at that time. (F, H, 6^th^ year, Nigeria)

### Theme 4.4: Other challenges

In addition to academic, sociocultural and wellbeing challenges, participants experienced other challenges such as food, climate, financial hardship, and dissatisfaction with scholarship evaluation. Vegetarian participants, especially from India, found it very hard to gain access to the food they wanted to eat. Some participants did not like Chinese food in the canteen and tried to cook by themselves. However, even cooking on one’s own was not easy as it took them time to go grocery shopping and to cook. Participants reported that in University A, cooking was forbidden inside the dorm due to fire control. There was one public kitchen located on the ground floor of their dorm building, but it was small and crowded, challenging for those who lived in higher floors to come down when there was no elevator installed inside that building.

Some participants from warm climates found winter in China very hard due to their lack of preparedness. In the first year, they came to China unprepared for winter, without having warm clothes or shoes. As a result, they easily caught a cold and/or a fever, and had breathing difficulties. Because of the cold weather, sometimes participants missed classes to stay in their dorm for warmth.

A number of participants (13%) also indicated that they experienced financial struggles due to their weak financial background, an unexpected family financial crisis (parents unemployed), or governmental changes in their home country. They were unable to pay fees in time or buy food, and experienced stress and weight loss.

At that time, my mind was going to a chaotic space, like what if my fees cannot be paid, but maybe I have to leave the college, maybe I cannot continue my MBBS studies. This tension, stress affected me a lot. (F, H, graduate, Bangladesh)

Some of the participants (8%) perceived that university scholarship evaluation mechanisms attached too much weight to extracurricular activity participation in proportion to academic grades. Those who received more activity credits but lower academic scores outranked those who who came top in academic grades in scholarship ranking. This perceived unfairness demotivated a few high-achievers who were scored high but lacked non-academic activity credit.

### Theme 4.5: Negative influence from teachers, administrators and classmates

In addition to teachers’ lack of English language ability and their teaching style (e.g. no interaction with students) as discussed earlier, participants also expressed their dissatisfaction with certain teachers for not managing class discipline or not checking attendance, lacking a passion for teaching, not caring about teacher-student rapport building, not updating the lecture slides (and making students read the slides for them), speaking in a low voice, or leaving immediately when a lecture had finished. This lack of interest and interaction from some teachers was demotivating and affected participants’ learning experience negatively:

During our internship, in the cardiology department, the department head was very busy. He did not want us to follow him. He said ‘you find someone else.’ ‘you go to another department, don’t come to me.’ I think that’s kind of cold. (M, L, graduate, India)

This dissatisfaction was detrimental to participants’ learning interest and focus, learning behaviors, engagement, and class attendance and behaviors. For example, participants might not be serious about a particular subject or delay learning that topic because of the teachers. Although participants learned by themselves when they felt they got little from class, it was time-consuming, and it was difficult for them to understand topics without teachers’ explanations. They indicated that their subject exam grades were adversely affected because of teachers:

When you have the same teacher, who doesn’t teach well, coming for five different classes. That’s a lot of content. Usually while we are in the class, we were not interested in the class. Sometimes it’s very boring. It is hard to concentrate. I have to study that topic by myself, but it’s sometimes very difficult because it’s hard to understand without somebody teaching you. So maybe you don’t do so good in those topics. (M, H, 6^th^ year, Australia)

Administrators could be a negative influence on students when a few situations occurred. A number of participants (23%) felt demoralized to study when some administrators did not follow up on their complaints about cheating in exam halls, showed favoritism to certain students without fairness, did not personally connect with students, could not understand students’ problems, or failed to give consideration to those students who were forced to be outside China during the pandemic:

I remember the most about people cheating in exams. There are cameras. Why can’t administrators always check and see who were cheating because it’s hard or very sad for the people who actually spend their time studying and then not getting enough scores. This is unfair. I personally felt this school could have done much more. (F, H, graduate, Bangladesh)

Some participants (10%) stated that their classmates could be discouraging when they interacted, disturbed others by talking and making noises, created negative competition, or made others feel less intelligent. Due to language barriers and social grouping between African and Indian students, some classmates of different nationalities might not relate much to each other:

Whenever you interact with a lot of classmates…as human beings, they demotivate you to study… they are like let’s go out, don’t study, let’s go eat outside. Those little things… (M, L, 5^th^ year, Ghana)

## Discussion

 This qualitative study explored international students’ motivation for choosing China to study medicine and their perceptions of academic success, and the factors they perceived to have affected their academic success. Concerning participants’ motives for studying medicine in China, the study found that the participants were “pushed” to seek opportunities outside their country and “pulled” to study in China primarily by educational cost considerations and by recommendation. High education fees are an important factor in deterring students, especially from developing countries, from studying abroad [45]. As tuition fees in the US, Australia, Europe, or Canada are higher than those in China, it is not surprising to see students’ choosing China as a country in which to pursue their studies.

Unlike in some studies, conducted in Germany [46], Australia [47], Sri Lanka [48], and Iran [49], participants’ conception of academic success in the present study encompassed more than just academic grades (the simple traditional indicator); it also included skills related to the practice of medicine, such as mastery of medical knowledge, clinical skills and communication skills. This finding has several implications. The first is that the participants were aware of the limitations of academic grades, in the sense that attaining good grades may mean that one is good at academic assessments and exams but does not necessarily mean that one has learned the practical skills necessary to practise medicine and help patients. This finding is in alignment with the literature on professions in the twenty-first century, and the requirements that employers are looking for in new professionals, in and outside health care systems [50]. The second is participants’ understanding of the need for clinical and communication skills that they can use to practice medicine in their national health systems. These findings suggest that Chinese medical universities hosting international students need to make some adjustments to their clinical training and experiential learning to provide these students with the skills that allow them to feel confident in practising medicine, and in doing so while facing the diseases that they are likely to encounter in their countries of origin.

Furthermore, this study identified a number of factors contributing to the academic success of the participants. These factors were consistent among both high-and low-achieving participants. Positive factors included support systems and campus resources, which is consistent with previous studies on social support [51–53], and campus support resources [30, 54]. In line with the findings of previous research studies conducted in different contexts, negative factors included academic issues [55, 56], sociocultural issues [57–59], wellbeing issues [60–62], and other challenges. The influence of teachers, administrators, and classmates could be both positive and negative.

In this specific context, the present study revealed salient differences. The findings indicated that a major area of concern for international students is Chinese teachers’ inadequate command of English. Studies have reported that the English language competence of faculty staff is positively correlated with international students’ GPA [63]. However, there are very few studies examining teachers’ English language competence in relation to international medical students’ academic results. This is probably due to the fact that most studies were conducted in English-speaking countries, where faculty teachers speak English as a first or official language. China is a non-English-speaking country, and the English language competency of its teachers is not explicitly known.

Furthermore, the findings revealed that teachers’ often limited English created a barrier that limited the ability of teachers to deliver information effectively through lectures, which may have negatively affected teachers’ teaching methods, teacher-student interaction and teacher support. Deficiences in these aspects tended to have a negative effect on international students’ learning. This confirms previous findings showing that teachers have a profound influence on international students’ learning in terms of their communication styles, teaching methods, available office hours, sympathy towards students, rapport building and so forth [19, 64]. Moreover, correlations have been found between teacher support and positive and negative academic emotions such as enjoyment or boredom [65]. Good teacher support directly and positively influences students’ motivation and self-efficacy, which positively affects student learning outcome [66]. In this sense, teaching quality is the key factor influencing the academic success of international medical students in China.

Another significant area of concern for Chinese hosting universities concerns English and Chinese language barriers for international students. Our findings confirm that international medical students’ English language is a critical factor in their academic success [33, 67, 68]. The findings in this study showed that the international student participants’ inadequate level of Chinese proficiency prevented them from communicating easily with Chinese patients and doctors during clinical lessons and internship [69], and also prevented them from socializing with local students. These findings highlight the need to improve the languages used when teaching in terms of both English and Chinese for international medical students throughout the degree program, and/or to include more stringent language requirements in either language or both languages for entry into the MBBS program.

In this study, adjusting to China’s medical education system (sequencing of curriculum, late exposure to clinics, limited clinical learning, poorly designed exams) caused difficulties and exam failure. The prior education experience of international students’ in their country of origin may affect their learning in a host institution [39], particularly if they are weak in natural science subjects. These findings are line with the literature suggesting that medical students encounter academic difficulties related to test-taking, test-anxiety, and organizing information [70]. In this respect, it may be helpful for Chinese medical universities to better prepare international students to survive and thrive with the curriculum, learning, and assessment requirements of the Chinese medical educaton system. Issues with internships are also worthy of attention. In line with previous Chinese studies [71, 72], participants’ academic learning of clinical and communication skills was hampered by a communication barrier between the participants and supervising doctors and patients, a lack of attention and care shown to students by staff, participants encountering diseases different from those in their home country, unfamiliar treatment practices, and social distance between Chinese patients and students. This issue seems to be a prevalent challenge for international students that Chinese universities need to address.

This study revealed that the mode of online teaching and learning, while having a few positive features such as flexibility in study time, was a poor substitute for learning in a traditional classroom setting. This is in consonance with the findings of recent studies showing that the majority of international medical students were dissatisfied with online classes at Chinese universities [73, 74]. Before China allows its international medical students back for face-to-face learning, hosting institutions should attach importance to enhancing the quality of their online teaching [74].

An important and new finding in this study was the influence of administrators and classmates. Administrators can have both a positive and a negative influence on students depending on their professionalism, work ethic, care and support for students. In this study, they affected participants’ overall academic and non-academic experiences, which indirectly affected their academic performance. Similarly, classmates, another important source of social support, seemed to have a long-term influence on each other’s daily lives and studies, as they spent almost the entire six years together in China. As a consequence, they were able to create a good competitive and collaborative academic environment.

There are several limitations to our findings. First, a few low-achieving students declined our interview invitation. Their non-participation in our study may have led to bias in the data analysis. Second, although the participants were recruited from two medical universities, the small sample size may limit the generalizability of the results. Third, the results of the study were only based on international medical students’ own views and reports.

## Conclusion

This study is, to the best of our knowledge, the first to investigate factors affecting the academic success of international medical students in China. The findings show that the factors influencing their academic success are multi-faceted. Ensuring the academic success of this group of students studying medicine in China is more of a collective responsibility, from the openness and inclusiveness of the host society, the quality of teachers, university services, campus resources, classmates, family, to students themselves. These findings reveal that Chinese universities have their own advantages for international students, such as a favorable campus environment, rich campus resources, universities services, and good teachers. However, much still needs to be done to help international students succeed in their studies. For example, medical education for international students may be improved if teachers become more fluent in English. Teaching pedagogy also seems to require some revisions and adaptations, and the curriculum needs to be reformed to cater to international standards and to address the challenges that these students will face when returning to their countries of origin. Extending the national medical school accreditation system to include international MBBS programs in China would require medical universities to review the qualifications, expertise, teaching pedagogy, and language proficiency of teachers in such programs, and if needed to implement improvements to meet the accreditation standards.

The findings suggest that the bar needs to be raised for the admission of international students in terms of English language proficiency and academic ability. It is important that students are well informed about, set aligned expectations, and prepared for the Chinese higher education system before arrival. The teaching and learning of the Chinese langage should be further strengthened during the course of the degree program to assure a more effective clinical learning. On campus, attention should be paid to recognizing and addressing students’ academic difficulties, sociocultural adjustment, wellbeing, and other challenges.

This study contributes to the literature by providing not only new insights into factors affecting the academic success of international medical students in a China setting but also information that can inform policy-makers and educators in developing policies that will improve the quality of international medical education in China. Future research is needed to explore Chinese teachers’ and administrators’ opinions on factors affecting international medical students’ academic success.

## Data Availability

The datasets generated and/or analysed during the current study are not publicly available due to privacy agreements between the researchers and the interviewees. Making the interviews available – considering the relatively low number of international students at both Medical Universities – would likely compromise the anonymity of the respondents, thus breaking the ethical consent agreement that was established. Requests for data can be considered and if considered reasonable are available from the corresponding author Hugo Horta (horta@hku.hk) or Qinxu Jiang (e-mail: jiangviolet86@hotmail.com).

## Footnotes

1 Internship is a term widely used in the Chinese health and higher education systems as well as in many other countries. In other parts of the world, including the US, it is known as “hospital residency”.

2 China has the greatest number of Indian students enrolled in MBBS programs compared with other popular countries including Russia, Ukraine, Nepal, Kyrgyzstan, Georgia, Bangladesh, the Philippines, Kazakhstan, Bangladesh, and Armenia.
